# Pitch Perception in Tone Language-Speaking Adults With and Without Autism Spectrum Disorders

**DOI:** 10.1177/2041669517711200

**Published:** 2017-06-05

**Authors:** Stella T. T. Cheng, Gary Y. H. Lam, Carol K. S. To

**Affiliations:** The University of Hong Kong, Hong Kong; University of South Florida Tampa, FL, USA; The University of Hong Kong, Hong Kong

**Keywords:** autism spectrum disorders, pitch perception, lexical tone, tone language

## Abstract

Enhanced low-level pitch perception has been universally reported in autism spectrum disorders (ASD). This study examined whether tone language speakers with ASD exhibit this advantage. The pitch perception skill of 20 Cantonese-speaking adults with ASD was compared with that of 20 neurotypical individuals. Participants discriminated pairs of real syllable, pseudo-syllable (syllables that do not conform the phonotactic rules or are accidental gaps), and non-speech (syllables with attenuated high-frequency segmental content) stimuli contrasting pitch levels. The results revealed significantly higher discrimination ability in both groups for the non-speech stimuli than for the pseudo-syllables with one semitone difference. No significant group differences were noted. Different from previous findings, post hoc analysis found that enhanced pitch perception was observed in a subgroup of participants with ASD showing no history of delayed speech onset. The tone language experience may have modulated the pitch processing mechanism in the speakers in both ASD and non-ASD groups.

The Fifth Edition of the Diagnostic and Statistical Manual of Mental Disorders (DSM-V) diagnoses autism spectrum disorders (ASD) on the basis of social communication and interaction deficits and restricted, repetitive behavior or interests (American Psychiatric Association, 2013). Although sensory abnormality belongs to one of the four diagnostic symptoms within the domain of restricted, repetitive behavior or interests in DSM-V (American Psychiatric Association, 2013), it is not a core diagnostic criterion for defining ASD. Leekam, Nieto, Libby, Wing, and Gould (2007) highlight the pervasiveness of sensory issues in children with ASD in various modalities, including sight, audition, olfaction, taste, and touch. Idiosyncratic sensory responses can be hyposensitive or hypersensitive, and many individuals with ASD show both response types within the same modality (Kern et al., 2006). In terms of auditory hyposensitivity, children with ASD may show no reaction to speech even though they have a normal hearing threshold (Grandin & Scariano, 1986). At the same time, they may exhibit auditory hypersensitivity to certain acoustic signals that manifest as distress.

Atypical auditory processing ability in ASD has been widely investigated. Two recent systematic reviews discussed in detail the behavioral, electrophysiological, and neuroanatomical evidence on atypical auditory processing in ASD compiled to date (Haesen, Boets, & Wagemans, 2011; O’Connor, 2012). For example, prior studies have reported atypical perception of pitch and loudness, orientation to auditory stimuli, prosody comprehension, and processing of auditory signals in noise in those with ASD (see O’Connor, 2012 for a review). Meanwhile, with respect to pitch perception in particular, reports of enhanced pitch discrimination ability in musically naïve children with ASD are not uncommon (e.g., Bonnel et al., 2003; Heaton, 2003, 2005; Heaton, Hudry, Ludlow, & Hill, 2008; Miller, 1989). For example, Bonnel et al. (2003) reported that children and adolescents with ASD generally outperformed their typically developing peers at discriminating and characterizing simple tones based on pitch. Similarly, Heaton, Davis, and Happé (2008) reported a case of an adult with ASD who demonstrated exceptional absolute pitch, which refers to the ability to identify the correct musical pitch (pitch classes or tone chromas) of isolated tones quickly with no external reference (Miyasaki, Makomaska, & Rakowski, 2012).

Järvinen-Pasley and Heaton (2007) directly tested whether enhanced auditory processing in ASD is domain-general or domain-specific. That is, whether children with ASD demonstrate similar sensitivity to pitch in both non-speech and speech stimuli or if they show disproportionately higher sensitivity to the former. They compared the pitch sequence discrimination ability of children with and without ASD, with speech and music pitch sequences used as the stimuli. The ASD group demonstrated similar performance across the three conditions, whereas the control group showed varying performance, with the speech-music condition the most challenging, followed by the speech-speech condition. The authors explained that the pitch information in the speech sequences may be relatively more salient to the ASD group than to the control group. It may also be the case that the control group was more influenced by the linguistic content in the speech sequences, whereas the ASD group was more capable of focusing on the pitch variation alone. The ASD group’s similar performance in both the music and speech conditions therefore led Järvinen-Pasley and Heaton (2007) to conclude that the enhanced auditory processing seen in ASD is domain-general.

The superior performance of individuals with ASD is often explained with reference to enhanced perceptual functioning (EPF) theory (Mottron & Burack, 2001; Mottron, Dawson, Soulières, Hubert, & Burack, 2006) and weak central coherence (WCC) theory (Happé & Frith, 2006). According to updated EPF theory (Mottron et al., 2006), perception in ASD is locally biased, rendering the individuals readier to engage in single dimensional processing such as pitch discrimination in the auditory modality or pattern recognition in the visual modality (Bertone, Mottron, Jelenic, & Faubert, 2005). As a result, ASD individuals exhibit an enhanced ability to process detailed information at the local level. In the EPF model, perception at the global level can be either intact or impaired. A stronger view of the atypical auditory perception observed in ASD is described by WCC theory, which highlights the difficulties at the global level that leads to the failure of the individuals with ASD to integrate information from various sources into a coherent whole because of their local bias (Happé & Frith, 2006). Järvinen-Pasley and Heaton (2007) explained the perceptual advantage of their ASD participants with reference to both theories. Those participants focused on pitch discrimination, as instructed, and ignored other aspects of the input, including verbal-semantic information. Thus, their pitch discrimination performance was unaffected by the semantic content, unlike the neurotypical (NT) controls.

Scholars have also highlighted the contribution of stimulus complexity during perception in ASD. Stimuli of different degrees of complexity require different levels of neuro-integrative processing and lead to varying performance in auditory processing in ASD (Bonnel et al., 2010; Samson, Mottron, Jemel, Belin, & Ciocca, 2006). Evidence of such differential neuro-integrating processing was initially put forward to describe visual perception performance in ASD (Bertone et al., 2005). In that original study, individuals with ASD demonstrated better control in identifying the orientation of first-order static visual stimuli than in tasks requiring second-order static visual stimuli, as the latter required more extensive neural circuitry. Samson et al. (2006) reanalyzed the behavioral and neurological data in the literature on auditory perception in ASD and concluded that individuals with these disorders exhibit a similar dissociation in the performance of auditory perception between simple and complex stimuli as in the visual modality. These researchers contended that the data support the neural complexity hypothesis, which posits that the perception of simple low-level stimuli is enhanced in ASD, whereas the perception of complex stimuli, which requires more neural resources, is impaired.

On the other hand, less robust findings or even reversed patterns of the auditory discrimination performance in ASD are also observed in the literature. For example, Heaton, William, Gummins, and Happé (2008) pointed out that individuals with ASD showing exceptional pitch discrimination skills are the minority (around 9%). In addition, the superior pitch processing skills of the three individuals with ASD was not related to their intelligence level with two of them demonstrating normal IQ and one demonstrating significant impairment. Jones et al. (2009) also reported similar findings that enhanced frequency discrimination was only exhibited by a subgroup (20%) of the adolescents with ASD. Unlike Heaton, William et al. (2008), Jones et al. found that most of the individuals with superior pitch perception skill belonged to the high IQ group (IQ > 80) and a number of them had a history of delayed speech onset.

In a more recent study, Kargas, López, Reddy, and Morris (2015) explored the presence of the enhanced low-level auditory discrimination skills in ASD with a more stringent research design such that the individuals with ASD were matched for age, IQ, and absence of formal musical training with non-ASD controls. Unlike most previous findings, Kargas et al. reported a generally diminished, rather than enhanced, performance in frequency discrimination in the ASD group when compared with the controls. However, exceptionally enhanced frequency discrimination skill was still observed in the group but only restricted to a small number of individuals with ASD (around 9%). Interestingly, the number of individuals in the non-ASD group showing enhanced pitch perception was similar (around 14%).

The stronger design of Kargas et al. (2015) provided a more complete picture regarding the observation of enhanced auditory perception in ASD after taking various potential contributing factors (e.g., functioning level, age of the individuals, and formal musical training) into account. In summary, enhanced pitch perception is observed in ASD but only happens in a subgroup of individuals rather than as a pervasive phenomenon across the population. In addition, the perceptual advantage is generally limited to low-level pitch detection or non-speech stimuli.

## Definition of Low-Level Stimuli in Tone Languages

**Figure 1.**
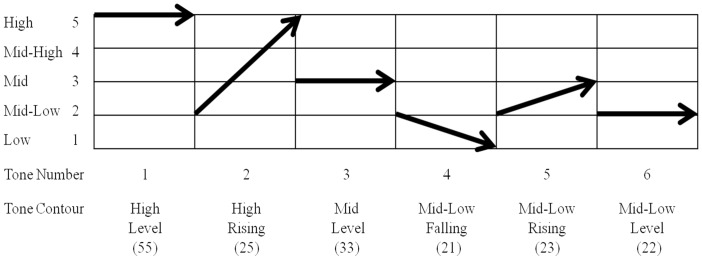
Cantonese lexical tones.

The distinction between low-level and complex auditory stimuli is relatively straightforward in non-tone languages, as pitch carries no linguistic information at the lexical level. However, that distinction is less clear-cut in tone languages, in which pitch variation within a syllable entails a change in lexical meaning. Cantonese is a typical example of tone languages and has six citation tones. Each syllable carries a lexical tone. For example, the Cantonese syllable /ji/ with a high-level (55) tone refers to “clothes,” whereas with a mid-level (33) tone it means “idea,” and with a mid-low level tone (22) it means “son.” In addition to different tone levels, there are also three contour tones: high-rising (25; /ji25/ means “chair”), low-rising (23; /ji23/ means “ear”), and low-falling (21; /ji21/ means “two”; Bauer & Benedict, 1997; see Figure 1). The tonal features of Cantonese provide unbiased and interesting grounds for exploring whether individuals with ASD who speak the language demonstrate auditory domain-specificity (Järvinen-Pasley & Heaton, 2007) and superior pitch perception at the local level, as predicted by EPF model.

The study reported herein addressed two research questions. First, how is pitch perception affected by the nature of auditory stimuli (i.e., real syllable, pseudo-syllable, and non-speech tones) in Cantonese-speaking adults with ASD? In other words, do Cantonese-speaking adults with ASD exhibit similar sensitivity to pitch in speech with meaning (real syllable), speech without meaning (pseudo-syllable), and non-speech stimuli? To find the answer, the performance of individuals with ASD was compared with that of a group of matched NT adults. Second, do the individuals with ASD exhibit a superior ability to detect subtle differences between pitch levels compared with their NT counterparts?

If the ASD group exhibited similar performance in response to real syllable, pseudo-syllable, and non-speech stimuli, then that finding would provide support for the reduced domain-specificity noted in Järvinen-Pasley and Heaton (2007). Based on EPF theory (Mottron et al., 2006), it was also hypothesized that the ASD group would be more sensitive to detecting differences in the stimuli at a local level. The meaning in speech stimuli was found to have an adverse effect on pitch discrimination in those without ASD (Heaton, Hudry et al., 2008; Järvinen-Pasley & Heaton, 2007). Hence, it was anticipated that the NT group in this study would perform significantly better in processing non-speech and pseudo-syllable stimuli than real syllable stimuli while the ASD group was unaffected by the nature of the stimuli.

## Method

### Participants

All participants were native Cantonese speakers who had received at least 9 years of compulsory education at mainstream schools in Hong Kong. Only those with no reported intellectual disabilities and with both normal hearing and normal visual acuity with or without correction were eligible for participation. The hearing ability of all participants was screened with a GSI 18 screening audiometer in a sound-proof booth at The University of Hong Kong. The passing criteria were set as at 25 dB HL at frequencies of 1000, 2000, and 4000 Hz in both ears (American Speech-Language-Hearing Association, 1997). Only those who passed the hearing screening were included.

#### ASD group

Participants in the ASD group were recruited from employment programs particularly designed for young adults who have been diagnosed with Asperger syndrome or high-functioning Autism. The employment programs were run by two local nongovernmental organizations in Hong Kong. The ASD diagnosis of the participants was made during their childhood by a clinical psychologist or a pediatrician. At that time in Hong Kong, clinicians mainly based their judgment on the clinical criteria stated in the Diagnostic and Statistical Manual of Mental Disorders, third edition (DSM-III; American Psychiatric Association, 1980) and/or International Classification of Diseases, 10th revision (ICD-10; World Health Organization, 1992). The current state of ASD was verified by the clinical judgment of the third author who is a speech-language pathologist with ASD expertise, and the Autism Diagnostic Observation Schedule (ADOS-2; Lord et al., 2012; Module 4) administered by research-reliable personnel. Twenty participants with ASD were recruited and passed the hearing screening. The mean age of the group was 25 years (*SD* = 3.22) ranging from 18 to 33 years old and 17 of them were males. Among the 20 participants, three participants scored at the range of “autism spectrum” and 17 participants at or above the thresholds of “autism” in the Module-4 of ADOS-2 (Lord et al., 2012). According to the parent reports, 3 participants in the ASD group produced the first words around their first birthday while 16 participants were reported to have a delayed onset of their first words ranging from 30 months to 4 years old (see Table 1). One participant failed to provide the information regarding his speech onset from his parents.

**Table 1.** Participant Characteristics of the ASD Group.

ADOS classification	Mean ADOS-2 (*SD*)	Mean onset of speech (*SD*)	Education		
			Secondary	Vocational training	Tertiary
Autism spectrum	7.33 (5.8)	1.83 years (1.44)	0	2	1
Autism	12.41 (1.97)	3.11 years (0.53)	2	9	6

ADOS = Autism Diagnostic Observation Schedule.

#### NT group

Twenty NT adults who met the inclusion criteria were recruited as the control group. The participants in the ASD group were matched individually with those in the NT group for sex, chronological age, and education background, as well as for the experience of formal musical training, which has been shown to have potential confounding effects on pitch perception ability (Schön, Magne, & Besson, 2004). Participants in the NT group filled out the questions from the Autism Spectrum Quotient (AQ; Baron-Cohen, Wheelwright, Skinner, Martin, & Clunbley, 2001). The average AQ scores of the NT group were 18.25 (*SD* = 5.48; range = 9 to 29) and none of the participants scored higher than the suggested cutoff of 32 (Baron-Cohen et al., 2001). The mean age of the NT group was 24 years (*SD* = 3.6) ranging from 17 to 34 years old.

Participants’ nonverbal IQ scores were estimated using the average score between two abbreviated nine-item forms of the Ravens Standard Progressive Matrices developed in Bilker et al. (2012). The two cohorts did not differ in Ravens scores (Mann–Whitney *U* = 141, *p* = .97).

### Stimuli

**Figure 2.**
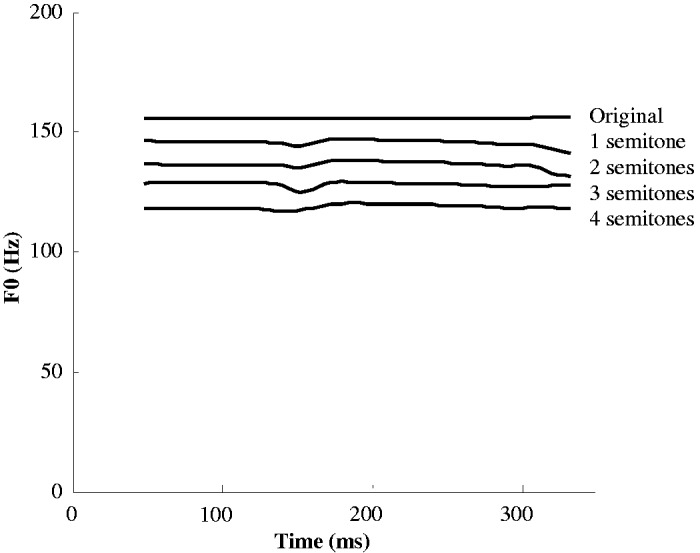
Fundamental frequencies of real syllable and pseudo-syllable stimuli.

The pitch discrimination experiment involved three types of auditory stimuli: real syllables, pseudo-syllables, and non-speech tones. Two monosyllabic words were used in each of the real syllable and pseudo-syllable conditions comprising a consonant-vowel-(consonant) combination with a fricative or glide and they were /jɐm/ and /sɛ/. Two pseudo-syllables /fam/ and /wɛ/, which exist either as a systematic gap that violates the phonotactic rules of Cantonese or an accidental gap, were used as the pseudo-syllable stimuli. These two types of stimuli were produced by a male native Cantonese speaker with Tone 1, that is, a high-level (55) tone in Cantonese. For each monosyllabic word, four stimuli with different pitches were synthesized from the original stimulus by shifting the pitch contour down by about one semitone in a stepwise manner using the PRAAT program (Boersma & Weenink, 2001). As a result, each monosyllabic word consisted of stimuli with five different pitches, as illustrated in Figure 2.

Twenty-five pairs of stimuli of different interval sizes (i.e., one semitone, two semitones, three semitones, four semitones, and no difference) were generated for each syllable, with the occurrence of the five tones counterbalanced. Two syllables were used in each speech stimulus condition, yielding 50 trials for each. For the non-speech stimuli, the value of fundamental frequency was extracted from the real syllable stimuli. Then, croaking-like non-speech stimuli were generated using PRAAT. The resulting stimuli were manipulated to produce 25 stimulus pairs, as for the speech stimuli. All pairs were repeated once, and thus the non-speech stimulus condition also had 50 trials.

### Procedures

An auditory discrimination task was employed as an experiment in which the stimulus pairs were presented by a computer. Stimuli were blocked according to type. Auditory instructions read aloud by the same speaker who recorded the stimuli were presented before the task. Within each block, stimuli were presented randomly using E-Prime 2.0 software. Two practice trials (one with identical pair and one with one-semitone difference) with feedback informing the participants of the correct answers were included in each block to provide a sense of the difference that required the participant to note. Within each pair, the inter-stimulus interval was around 700 ms. When a pair of stimuli was presented, the participants were asked to indicate whether the stimuli were the same or different by pressing the corresponding keys on the keyboard. The number of correct responses was captured and recorded by the E-Prime program. The blocks were presented in an order that was counterbalanced across participants from the same group. Each participant was tested individually, and all experiments took place in a quiet room.

## Results

All data from the pitch discrimination task were recorded in the E-Prime program and then imported to the SPSS program for statistical analysis. Descriptive data on the results of the different stimuli types in the pitch discrimination task are displayed in Table 2. It can be seen that, in general, all participants performed better in the non-speech stimulus condition, followed by the pseudo-syllable and then real syllable conditions, except for the interval with no difference. As expected, the larger the pitch interval size, the higher the degree of accuracy in the discrimination task. Both the ASD and NT groups displayed close to perfect performance in discriminating the pairs in which the syllables differed by four semitones.

**Table 2.** Mean Percentages of Correct Trials Across Stimulus Conditions (*SD*s in Parentheses).

Group	Tone interval size				
	1 semitone	2 semitones	3 semitones	4 semitones	No difference
Real syllable				
ASD	62.19 (29.42)	89.17 (16.91)	95.00 (13.08)	95.00 (22.36)	96.00 (6.81)
NT	68.88 (23.1)	92.08 (16.77)	96.88 (8.95)	98.75 (5.590)	97.50 (6.39)
Overall	65.47 (26.32)	90.63 (16.69)	95.94 (11.10)	96.88 (16.20)	96.75 (6.56)
Pseudo-syllable					
ASD	61.88 (33.50)	90.42 (17.58)	94.38 (11.09)	95.00 (10.26)	92.5 (10.2)
NT	62.50 (29.73)	95.00 (13.08)	96.88 (11.38)	96.25 (12.23)	93.5 (9.88)
Overall	62.19 (1.26)	92.71 (15.47)	95.63 (11.16)	95.62 (11.16)	96.25 (7.40)
Non-speech					
ASD	70.31 (26.67)	92.08 (12.53)	95.63 (12.35)	92.50 (20.03)	92.50 (10.20)
NT	70.31 (25.88)	93.33 (13.41)	94.38 (16.95)	96.25 (12.23)	93.50 (9.88)
Overall	70.31 (25.93)	92.71 (12.83)	95.00 (14.65)	94.38 (16.49)	93.00 (9.23)

*Note*: ASD = autism spectrum disorders, NT = neurotypical controls.

Data were analyzed using a three-way mixed-effect model analysis of variance (ANOVA) model, with group (ASD vs. NT) as the between-subject factors and stimulus type (real syllables, pseudo-syllables, vs. non-speech sounds) and interval size (one, two, three, vs. four semitones) as the within-subject factors. The percentage of correct trials at interval size in the different stimulus types served as the dependent variable.

The assumption of sphericity was violated for stimulus types χ*^2^*(2) = 6.72,* p* = .035, interval size, χ*^2^*(9) = 77.09, *p* < .001, and the interaction of these two within-subject factors, χ*^2^*(35) = 125.38,* p* < .001. The degrees of freedom were therefore corrected using Greenhouse–Geisser estimates of sphericity (ɛ = 0.85, ɛ = 0.51, and ɛ = 0.56 for stimulus type, interval size, and their interaction, respectively).

**Figure 3.**
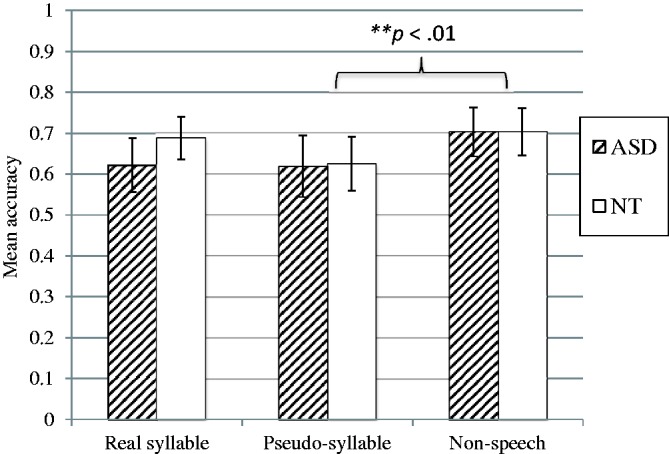
Effect of stimulus types at 1 semitone difference.

There was no significant main effect for stimulus type and no significant main effect for group, and the effect for stimulus type-by-group interaction was also not significant. On the other hand, the main effect of interval size was significant, *F*(1.46, 55.43) = 60.34, *p < *.001, $\eta_{p}^{2}$ = .641, with participants scoring significantly lower for the pairs with one semitone difference than for the others. In addition, the stimulus type-by-interval size interaction was also significant, *F*(6, 126.57) = 3.24, *p = *.004, $\eta_{p}^{2}$ = .079. Follow-up ANOVAs were conducted separately for each interval size. The follow-up analysis revealed the main effect of stimulus type to be significant only for the pairs with one semitone difference, *F*(1.85, 70.10) = 3.842, *p* = .029, $\eta_{p}^{2}$ = .092, but not those on other levels. Pairwise comparisons adjusted by the Bonferroni method showed the percentage of correct trials to be significantly higher in the non-speech stimulus condition (*M* = 70.31, *SD* = 25.93) than in the pseudo-syllable (*M* = 61.88, *SD* = 33.50, *p* = .007) while the differences between other stimulus condition pairs (real syllable vs. non-speech and real syllable vs. pseudo-syllable) were not significant. Figure 3 summarizes the results.

In summary, the participants in both the ASD and NT groups had significantly more correct trials for the stimuli in the non-speech than for those in the pseudo-syllable stimuli in discriminating pairs with one semitone difference but not for the other levels. The performance of the two groups was not significantly different. The ASD group did not exhibit enhanced pitch discrimination ability relative to the NT group regardless of the stimulus types.

### Subgroup Analysis

**Figure 4.**
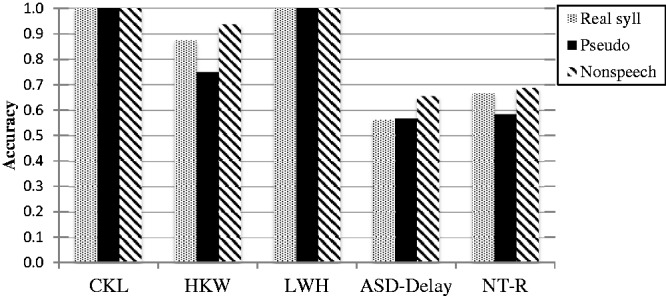
Performance of the CKL, HKW, and LWH in different stimulus types at 1 semitone difference comparing with the group performance.

Given the evidence of subgroup performance in pitch perception in ASD with reference to their onset of speech, a post hoc analysis focusing on the interval size of one semitone difference was conducted by separating the performance of the three participants with ASD showing no delay in their onset of speech (CKL, HKW, and LWH) and their group as well as their matched control group. Their performances were compared against the average group performances of ASD-Delay and NT-R, the groups after excluding them and their matched controls in the ASD and NT groups, respectively (see Figure 4). The three participants who showed no delay in the onset of their first words demonstrated heightened performance in general. The participants CKL and LWH showed perfect performance in all three stimulus types and HKW showed 0.93, 0.56, and 0.94 standard deviation above the mean of the NT-R group in the stimuli of real syllable, pseudo syllable, and non-speech, respectively. After excluding the six participants, the main effect of group and stimulus type, and their interaction effect remained the same such that only the main effect of stimulus type was significant, *F*(2, 31) = 5.79, *p = *.007, $\eta_{p}^{2}$ = .252. Pairwise comparisons adjusted by the Bonferroni method showed the percentage of correct trials to be significantly higher in the non-speech (*M* = 67.10, *SD* = 25.81) than pseudo syllable stimulus condition (*M* = 57.54, *SD* = 31.11).

## Discussion

The aim of this study was to examine whether Cantonese-speaking adults with ASD have a similar degree of sensitivity to pitch in speech (real syllables) and non-speech stimuli at monosyllable level when compared with matched controls. The ASD group demonstrated significantly weaker performance in discriminating the pitch pairs for the pseudo-syllable stimuli than non-speech stimuli and the differences between real syllable and non-speech, real syllable and pseudo-syllable were not significant. More specifically, the effect of stimulus type lay in the one semitone difference condition. The same pattern of better performance in non-speech and real syllable conditions than pseudo-syllable was also observed in the NT group.

The performance in the real syllable conditions revealed that the pitch processing involved tapped into the phonological representations of the participants in both groups. The difference of the two tones presented at two-semitone interval or more appeared to be sufficient for the listeners to encode another lexical tone category in the language as the accuracy was around 90% or above. When the categorical boundaries of lexical tones in the adults were intact, the discrimination of these pairs would not be a challenge. The patterns were not surprising as participants in the ASD group were able to communicate with verbal language in which lexical tones are the basic units. Discriminating units that contrast with meaning would not be a problem to them. When the difference of the two tones dropped to one semitone, the accuracy in both ASD and NT groups decreased quite remarkably, suggesting that the one semitone difference may be at the border of phonetic boundary for two lexical tones.

The nonsignificant difference in the performance between the real syllable and non-speech stimuli in the ASD group apparently supported the reduced domain specificity of pitch perception in autism described by Järvinen-Pasley and Heaton (2007). Their ASD group exhibited comparable discrimination skills in pitch sequence pairs in both music and speech conditions. The reduced domain specificity in the ASD group was explained by the participants’ exceptional ability in directing attention to smaller units of information regardless of contexts. In the present study, however, the same perceptual pattern was also noted in the NT control group, and the ASD participants as a group did not show enhanced or diminished performance. The presumably distracting linguistic information in the real syllable stimuli did not pose particular difficulty to both groups.

Strictly speaking, this study did not replicate the phenomenon that individuals with ASD mainly focus on low-level characteristics of sounds using the current paradigm. The present results only supported the claim that enhanced perceptual processing in ASD, when compared with non-ASD counterparts, may be mainly limited to low-level simple pure tone processing (e.g., Bonnel et al., 2003; Heaton, 2003; Heaton, Hudry et al., 2008; Lepistö et al., 2008; Mottron et al., 2006; O’Riordan & Passetti, 2006) but not spectrally complex stimuli as those used in the present study (Samson et al., 2011). The non-speech stimuli that modulated from speech stimuli in the present study were assumed to be low level in Cantonese because of the removal of the linguistic information. However, acoustically, the stimuli were spectrally complex when compared with pure tones. Therefore, enhanced performance in ASD may not be revealed in this case. In other words, stimuli complexity may still be a potential factor that contributes to the unusual auditory processing in ASD (Samson et al., 2011). More systematic and direct investigation of this speculation can be conducted.

Another possible explanation for the absence of atypical performance of the ASD group might be that enhanced perception in ASD is not determined by sound complexity. Čeponienė et al. (2003) suggested that the main problems of the children with high-functioning ASD lie in *selective attention* to “speech-like” stimuli; rather than at the sensory processing stage. By using event-related brain potentials evidence, Čeponienė et al. (2003) attested that children with high-functioning ASD showed typical brain responses in detecting pitch changes in speech and non-speech sounds but impaired involuntary attentional responses specific to speech sounds. The authors concluded that the perception of the different types of sound in children with autism at sensory processing stage is intact. However, they show deficit orienting to stimuli which is contingent on the speech likeness of the sound stimuli instead of acoustic complexity in general. Finally, both groups performed weaker in the pseudo-syllable stimuli than the non-speech stimuli. This observation was not of expectation. It might be possible that the pseudo-syllable stimuli contain more irrelevant information that interfered individuals’ processing. As a result, both groups found it more difficult to discriminate pitch in the intermediate speech and non-speech context.

The post hoc subgroup analysis after separating individuals with and without delayed onset of speech in the ASD group provided some additional information regarding the processing mechanism of the ASD group. The participants *with* delayed speech onset as a group (*n* = 16) in the present study showed no significant difference from their matched non-ASD controls in discriminating pitches in real syllables and non-speech. On the other hand, the three participants *without* delayed speech onset showed relatively stronger pitch discrimination skills in the three stimulus types when compared with the typical control group, and in particular the real syllables (see Figure 4).

The diverse performance of the participants with and without delayed speech onset was generally congruent to the observed heterogeneity of ASD in auditory perception described in previous studies (Bonnel et al., 2010; Heaton, Williams et al., 2008; Jones et al., 2009; Kargas et al., 2015). For examples, Jones et al. (2009) found that their adolescents with ASD in general as a group showed no processing advantage in detecting differences in frequency, and enhanced pitch discrimination was observed in a small proportion of the participants. Similarly, Bonnel et al. (2010) found that enhanced pitch discrimination for simple tones were only observed in their participants with autism, who exhibited a delayed onset of first words, but not with Asperger syndrome, who showed mostly typical milestones of speech characteristics (DSM-IV, APA, 1994). Heaton, Williams et al. (2008) and Kargas et al. (2015) provided the evidence that superior pitch processing ability is restricted to a subgroup of the ASD population rather than a pervasive phenomenon. In the present study, enhanced pitch discrimination was also observed as a characteristic of a particular subgroup of ASD individuals who speak Cantonese as their first language.

The relatively superior pitch perception ability of three individuals having no speech onset delay in real syllables may be due to the fact that they dedicated more neural resources to the processing of these stimuli. Samson, Zeffiro, Doyon, Benali, and Mottron (2015) found that their participants with ASD with no speech onset delay demonstrated greater task-related neural activity than typical individuals in several brain regions when processing non-social stimuli and frequency modulated sounds. Those highly activated brain regions included the inferior frontal gyrus (IFG), peri-auditory middle, and superior temporal gyri, which are associated with language processing. For those with speech delay, higher activity was also reported but mainly in the perceptual cortex, which is associated with low-level auditory cortical processing. Given that the stimulus materials used possess speech-like acoustic properties, the large engagement of language-related regions during pitch processing in the no-delay group may explain why this group also performed better when processing acoustic components that are relevant to speech recognition than the typical matched controls (Samson et al., 2015).

In addition, the findings might point to the language-specific influence on pitch perception in both typical and clinical population. Pitch perception in Cantonese speakers has a heavy functional load where speakers have to process lexical tones extensively in everyday life. The impact of this demand is reflected in studies that showed a language-specific pattern to tone perception as early as 4 months old in infants learning a tone language (e.g., Yeung, Chen, & Werker, 2013) and the association between pitch perception ability and children’s later language development (Antoniou, To, & Wong, 2015). Ample evidence has supported that linguistic experience can shape the functional circuit involved in pitch processing during speech perception. For example, significant differences in behavioral and neural responses to pitch in tone versus nontone language speakers in both speech and musical contexts had been reported (e.g., Giuliano, Pfordresher, Stanley, Narayana, & Wicha, 2011; Pfordresher & Brown, 2009; Wong, Parsons, Martinez, & Diehl, 2004; Wong et al., 2012). In particular, cortical processing of lexically relevant pitch contrasts in tone speakers is language specific (Hsieh, Gandour, Wong, & Hutchins, 2001; Wong et al., 2004). The linguistic functions of pitch may have modulated individuals’ perception mechanism in general. Cantonese-speaking individuals with ASD who showed some sensitivity to the language in the early years (i.e., without delayed onset of speech) may therefore possess an advantage in pitch processing after the long-term exposure to lexical tones. They therefore exhibited superior pitch perception ability in non-speech as well as speech materials. It may also be possible that the perception of pitch in individuals with ASD speaking tone and non-tone languages involves distinct processing mechanisms and different brain regions or pathways. Future brain studies would provide neurophysiological evidence to better supplement and account for these predictions.

The present finding provided certain new observations about pitch perception in ASD, but the study has several limitations. First, the present study only examined pitch discrimination. Previous studies suggested that the enhanced perception in ASD may be better realized in identification than discrimination task (Samson et al., 2006) and future studies may explore other testing methods to elicit the underlying skills. Second, the subgroup analysis is not an a priori study design. The number of participants with and without delayed speech onset was not even, which did not support powerful comparison with inferential statistics. Third, only speakers speaking one tone language were examined. Future studies can also involve individuals with other tone language background (e.g., Mandarin) to examine if similar phenomena can be observed. Finally, the present study made use of participants’ education level and their performance in the abbreviated form of the Raven’s Standard Progressive Matrices Test as the measures of the matching participants’ intelligence. Future studies could use IQ scores in Wechsler IQ test or the full version of the Raven’s Progressive Matrices.

## References

[bibr1-2041669517711200] American Psychiatric Association (1980) Diagnostic and statistical manual of mental disorders, (3rd edn.) Washington, DC: Author.

[bibr2-2041669517711200] American Psychiatric Association (2013) Diagnostic and statistical manual of mental disorders, (5th edn.) Washington, DC: Author.

[bibr3-2041669517711200] American Speech-Language-Hearing Association. (1997). *Guidelines on audiological screening*. Rockville, MD: Author.

[bibr4-2041669517711200] Antoniou M. To C. K. S. Wong P. C. M. (2015) Auditory cues that drive language development are language specific: Evidence from Cantonese. Applied Psycholinguistics 36: 1493–1507.

[bibr5-2041669517711200] Baron-Cohen S. Wheelwright S. Skinner R. Martin J. Clunbley E. (2001) The autism-spectrum quotient (AQ): Evidence from Asperger syndrome/high-functioning autism, males and females, scientists and mathematicians. Journal of Autism and Developmental Disorders 31: 5–17. 11439754 10.1023/a:1005653411471

[bibr6-2041669517711200] Bauer R. S. Benedict P. K. (1997) Modern Cantonese phonology, Berlin, Germany: Mouton de Gruyter.

[bibr7-2041669517711200] Bertone A. Mottron L. Jelenic P. Faubert J. (2005) Enhanced and diminished visuo-spatial information processing in autism depends on stimulus complexity. Brain 128: 2430–2441. 15958508 10.1093/brain/awh561

[bibr8-2041669517711200] Bilker W. B. Hansen J. A. Brensinger C. M. Richard J. Gur R. E. Gur R. C. (2012) Development of abbreviated nine-item forms of the Raven’s Standard Progressive Matrices Test. Assessment 19: 354–369. 22605785 10.1177/1073191112446655 PMC4410094

[bibr9-2041669517711200] Boersma P. Weenink D. (2001) PRAAT, a system for doing phonetics by computer. Glot International 5: 341–345.

[bibr10-2041669517711200] Bonnel A. McAdams S. Smith B. Berthiaume C. Bertone A. Ciocca V. Mottron L. (2010) Enhanced pure-tone pitch discrimination among persons with autism but not Asperger syndrome. Neuropsychologia 48: 2465–2475. 20433857 10.1016/j.neuropsychologia.2010.04.020

[bibr11-2041669517711200] Bonnel A. Mottron L. Peretz I. Trudel M. Gallun E. Bonner A. M. (2003) Enhanced pitch sensitivity in individuals with autism: A signal detection analysis. Journal of Cognitive Neuroscience 15: 226–235. 12676060 10.1162/089892903321208169

[bibr12-2041669517711200] Čeponienė R. Lepistö T. Shestakova A. Vanhala R. Alku P. Näätänen R. Yaguchi K. (2003) Speech-sound-selective auditory impairment in children with autism: The can perceive but do not attend. Proceedings of the National Academy of Sciences of the United States of America 100: 5567–5572. 12702776 10.1073/pnas.0835631100 PMC154385

[bibr13-2041669517711200] Giuliano R. J. Pfordresher P. Q. Stanley E. M. Narayana S. Wicha N. Y. Y. (2011) Native experience with a tone language enhances pitch discrimination and the timing of neural responses to pitch change. Frontiers in Psychology 2: 146. 21886629 10.3389/fpsyg.2011.00146 PMC3155092

[bibr14-2041669517711200] Grandin T. Scariano M. M. (1986) Emergence: Labeled autistic, New York, NY: Warner Books.

[bibr15-2041669517711200] Haesen B. Boets B. Wagemans J. (2011) A review of behavioral and electrophysiological studies on auditory processing and speech perception in autism spectrum disorders. Research in Autism Spectrum Disorders 5: 701–714.

[bibr16-2041669517711200] Happé F. Frith U. (2006) The weak coherence account: Detail-focused cognitive style in autism spectrum disorders. Journal of Autism and Developmental Disorders 36: 5–25. 16450045 10.1007/s10803-005-0039-0

[bibr17-2041669517711200] Heaton P. (2003) Pitch memory, labeling and disembedding in autism. Journal of Child Psychology and Psychiatry 44: 543–551. 12751846 10.1111/1469-7610.00143

[bibr18-2041669517711200] Heaton P. (2005) Interval and contour processing in autism. Journal of Autism and Development Disorders 35: 787–793. 10.1007/s10803-005-0024-7 16283085

[bibr19-2041669517711200] Heaton P. Davis R. E. Happé F. G. E. (2008) Research note: Exceptional absolute pitch perception for spoken words in an able adult with autism. Neuropsychologia 46: 2095–2098. 18358502 10.1016/j.neuropsychologia.2008.02.006

[bibr20-2041669517711200] Heaton P. Hudry K. Ludlow A. Hill E. (2008) Superior discrimination of speech pitch and its relationship to verbal ability in autism spectrum disorders. Cognitive Neuropsychology 25: 771–782. 18720290 10.1080/02643290802336277

[bibr21-2041669517711200] Heaton P. Williams K. Cummins O. Happé F. (2008) Autism and pitch processing splinter skills: A group and subgroup analysis. Autism 12: 203–219. 18308768 10.1177/1362361307085270

[bibr22-2041669517711200] Hsieh L. Gandour J. Wong D. Hutchins G. D. (2001) Functional heterogeneity of inferior frontal gyrus Is shaped by linguistic experience. Brain and Language 76: 227–252. 11247643 10.1006/brln.2000.2382

[bibr23-2041669517711200] Järvinen-Pasley A. Heaton P. (2007) Evidence for reduced domain-specificity in auditory processing in autism. Development Science 10: 786–793. 10.1111/j.1467-7687.2007.00637.x 17973796

[bibr24-2041669517711200] Jones C. R. G. Happé F. Baird G. Simonoff E. Marsden A. J. S. Tregay J. Charman T. (2009) Auditory discrimination and auditory sensory behaviours in autism spectrum disorders. Neuropsychologia 47: 2850–2858. 19545576 10.1016/j.neuropsychologia.2009.06.015

[bibr25-2041669517711200] Kargas N. López B. Reddy V. Morris P. (2015) The relationship between auditory processing and restricted, repetitive behaviors in adults with autism spectrum disorders. Journal of Autism and Developmental Disorders 45: 658–668. 25178987 10.1007/s10803-014-2219-2

[bibr26-2041669517711200] Kern J. E. Trivedi M. H. Garver C. R. Grannemann B. D. Andrews A. A. Savla J. S. Schroeder J. L. (2006) The pattern of sensory processing abnormalities in autism. Autism 10: 480–494. 16940314 10.1177/1362361306066564

[bibr27-2041669517711200] Leekam S. R. Nieto C. Libby S. J. Wing L. Gould J. (2007) Describing the sensory abnormalities of children and adults with autism. Journal of Autism and Developmental Disorders 37: 894–910. 17016677 10.1007/s10803-006-0218-7

[bibr28-2041669517711200] Lepistö T. Kajander M. Vanhala R. Alku P. Huotilainen M. Näätänen R. Kujala T. (2008) The perception of invariant speech features in children with autism. Biological Psychology 77: 25–31. 17919805 10.1016/j.biopsycho.2007.08.010

[bibr29-2041669517711200] Lord C. Rutter M. DiLavore P. C. Risi S. Gotham K. Bishop S. (2012) Autism diagnostic observation schedule, (2nd ed.) Torrance, CA: Western Psychological Service.

[bibr30-2041669517711200] Miller L. K. (1989) Musical savants: Exceptional skills in the mentally retarded, Hillsdale, MI: Laurence Erlbaum.

[bibr31-2041669517711200] Miyasaki K. Makomaska S. Rakowski A. (2012) Prevalence of absolute pitch: A comparison between Japanese and Polish music students. The Journal of the Acoustical Society of America 132: 3484–3493. 23145628 10.1121/1.4756956

[bibr32-2041669517711200] Mottron L. Burack J. A. (2001) Enhanced perceptual functioning in the development of autism. In: Burack J. A. Charman T. Yirmiya N. Zelazo P. R. (eds) The development of autism: Perspectives from theory and research, Mahwah, NJ: Lawrence Erlbaum, pp. 131–148.

[bibr33-2041669517711200] Mottron L. Dawson M. Soulières I. Hubert B. Burack J. A. (2006) Enhanced perceptual functioning in autism: An update, and eight principles of autistic perception. Journal of Autism and Developmental Disorders 36: 27–43. 16453071 10.1007/s10803-005-0040-7

[bibr34-2041669517711200] O’Connor K. (2012) Auditory processing in autism spectrum disorder: A review. Neuroscience and Biobehavioral Reviews 36: 836–854. 22155284 10.1016/j.neubiorev.2011.11.008

[bibr35-2041669517711200] Pfordresher P. Q. Brown S. (2009) Enhanced production and perception of musical pitch in tone language speakers. Attention, Perception, & Psychophysics 71: 1385–1398. 10.3758/APP.71.6.1385 19633353

[bibr36-2041669517711200] Samson F. Hyde K. L. Bertone A. Soulières I. Mendrek A. Ahadc P. Zeffiro T. A. (2011) Atypical processing of auditory temporal complexity in autistics. Neuropsychologia 49: 546–555. 21192958 10.1016/j.neuropsychologia.2010.12.033

[bibr37-2041669517711200] Samson F. Mottron L. Jemel B. Belin P. Ciocca V. (2006) Can spectro-temporal complexity explain the autistic pattern of performance on auditory tasks? Journal of Autism and Developmental Disorders 36: 65–76. 16382329 10.1007/s10803-005-0043-4

[bibr38-2041669517711200] Samson F. Zeffiro T. A. Doyon J. Benali H. Mottron L. (2015) Speech acquisition predicts regions of enhanced cortical response to auditory stimulation in autism spectrum individuals. Journal of Psychiatric Research 68: 285–292. 26037888 10.1016/j.jpsychires.2015.05.011

[bibr39-2041669517711200] Schön D. Magne C. Besson M. (2004) The music of speech: Music training facilitates pitch processing in both music and language. Psychophysiology 41: 341–349. 15102118 10.1111/1469-8986.00172.x

[bibr40-2041669517711200] World Health Organization (1992) The ICD-10 classification of mental and behavioural disorders: Clinical descriptions and diagnostic guidelines, Geneva, Switzerland: Author.

[bibr41-2041669517711200] Wong P. C. M. Ciocca V. Chan A. H. D. Ha L. Y. Y. Tan L.-H. Peretz I. (2012) Effects of culture on musical pitch perception. PLoS One 7: e33424. 22509257 10.1371/journal.pone.0033424 PMC3324485

[bibr42-2041669517711200] Wong P. C. M. Parsons L. M. Martinez M. Diehl R. L. (2004) The role of the insular cortex in pitch pattern perception: The effect of linguistic contexts. Journal of Neuroscience 24: 9153–9160. 15483134 10.1523/JNEUROSCI.2225-04.2004 PMC6730056

[bibr43-2041669517711200] Yeung H. H. Chen K. H. Werker J. F. (2013) When does native language input affect phonetic perception? The precocious case of lexical tone. Journal of Memory and Language 68: 123–139.

